# The Influence of Different Irradiation Regimens on Inflammation and Vascularization in a Random-Pattern Flap Model

**DOI:** 10.3390/jpm13101514

**Published:** 2023-10-21

**Authors:** Wibke Müller-Seubert, Patrick Ostermaier, Raymund E. Horch, Luitpold Distel, Benjamin Frey, Ramona Erber, Andreas Arkudas

**Affiliations:** 1 Laboratory for Tissue Engineering and Regenerative Medicine, Department of Plastic and Hand Surgery, University Hospital Erlangen, Friedrich Alexander University Erlangen-Nuernberg (FAU), 91054 Erlangen, Germany; patrick.ostermaier@fau.de (P.O.); raymund.horch@uk-erlangen.de (R.E.H.);; 2 Department of Radiation Oncology, University Hospital Erlangen, Friedrich Alexander University Erlangen-Nuernberg (FAU), 91054 Erlangen, Germany; luitpold.distel@uk-erlangen.de; 3 Translational Radiobiology, Department of Radiation Oncology, University Hospital Erlangen, Friedrich-Alexander-University Erlangen-Nuernberg (FAU), 91054 Erlangen, Germany; benjamin.frey@uk-erlangen.de; 4 Institute of Pathology, University Hospital Erlangen, Friedrich Alexander University Erlangen-Nuernberg (FAU), Comprehensive Cancer Center Erlangen-EMN, 91054 Erlangen, Germany; ramona.erber@uk-erlangen.de

**Keywords:** irradiation, random-pattern flaps, vascularization

## Abstract

Background: Irradiation plays an important role in the oncological treatment of various tumor entities. The aim of the study was to investigate the influence of different irradiation regimens on random-pattern flaps at the molecular and histopathological levels. Methods: Twenty-five rats underwent harvesting of bilateral random-pattern fasciocutaneous flaps. The right flaps received irradiation, while the left flaps served as non-irradiated intraindividual controls. Five rats served as a non-irradiated control group. Four different irradiation regimens with give rats each were tested: 20 Gy postoperatively, 3 × 12 Gy postoperatively, 20 Gy preoperatively, and 3 × 12 Gy preoperatively. Two weeks after surgery, HE staining and immunohistochemical staining for CD68 and ERG, as well as PCR analysis to detect Interleukin 6, HIF-1α, and VEGF, were performed. Results: A postoperative cumulative higher dose of irradiation appeared to result in an increase in necrosis, especially in the superficial layers of the flap compared to preoperative or single-stage irradiation. In addition, we observed increased expression of VEGF and HIF-1α in all irradiation groups. Conclusion: Even though no statistically significant differences were found between the different groups, there was a tendency for fractional postoperative irradiation with a higher total dose to have a more harmful effect compared to preoperative or single-dose irradiation.

## 1. Introduction

The development of innovative methods by which to enhance tissue viability is one of the main goals of reconstructive surgery and tissue engineering. Defect reconstruction, especially after tumor resection using local or free flaps, is one of the daily challenges of a plastic surgeon. Total or partial flap necrosis has far-reaching negative consequences such as flap loss. Immediate reconstruction after tumor resection is indicated in some cases; for example, in patients with breast cancer. These patients benefit from a possible better aesthetic outcome as the skin envelope is preserved. Furthermore, delayed surgery is not necessary, and there are psychological benefits for the patients [1]. When immediate reconstruction is performed in advanced stages, postoperative radiotherapy including the transplanted flap might be indicated. Furthermore, irradiation is a standard adjuvant treatment for many tumor entities, intended to reduce the local risk of recurrence [2]. Postoperative irradiation is typically given following an allocated period of time to allow for convalescence and early wound healing, but not so long as to decrease its effectiveness because of microscopic tumor regrowth [3]. However, irradiation can possibly influence vascular and cellular mechanisms that are important, especially for local flap survival. Atrophia and hypovascularity are damaging side effects on the surrounding tissue [2]. In general, both adjuvant and neoadjuvant radiation are associated with significant morbidity caused by impaired wound healing, ulcers, or osteonecrosis with subsequent fractures [4]. Ionizing irradiation generates highly reactive chemical products such as free ion radicals. These free ion radicals can combine with normal body chemicals and react with cellular components, ultimately causing intracellular and molecular damage [5]. The tissue damage can result in dermatitis, necrosis, and delayed wound healing [5]. Irradiation of flaps, for example, after breast reconstruction, might result in hyperpigmentation of the skin, flap contracture, palpable fat necrosis, and reduction in flap volume [1,6]. One of its dose-limiting complications is the development of tissue fibrosis, which is associated with an increase in collagen synthesis and with the remodeling and sequential activation of key fibrogenic growth factors and cytokines [7]. Inflammation seems often to be needed for the initiation of fibrosis [7]. The inflammation results in the activation of aberrant cytokine pathways or the chronic overproduction of certain cytokines [8]. These processes result in uncontrolled matrix accumulation and the development of fibrosis [8]. Acute irradiation damage occurs mainly in tissues with rapid proliferating cells such as the skin or the epithelium of the gastrointestinal tract [9]. Interleukin-6 (IL-6), a prototypical cytokine that activates the acute phase and immune response, is immediately produced by immune cells such as macrophages and monocytes in response to stresses such as infection or tissue injury [10,11]. Moreover, inflammation can induce angiogenesis. Vascular endothelial growth factor (VEGF) is an angiogenic factor that is important for vascular development and maintenance in all mammalian organs [12]. Furthermore, it plays a key role in inflammatory angiogenesis [12]. VEGF promotes survival, induces proliferation, and enhances migration and invasion of endothelial cells, which contribute to angiogenesis [13]. In addition, hypoxia is a major inducer of VEGF gene transcription [14].

If hypovascularity or malperfusion occur, tissue response to low oxygen tension is initiated. This reaction is mediated by hypoxia-inducible factor-1 (HIF-1). Therefore, different genes that participate in angiogenesis, iron metabolism, glucose metabolism, and cell proliferation/survival are induced. HIF-1 consists of two subunits: the constitutively expressed subunit HIF-1β and the oxygen-regulated subunit HIF-1α. In hypoxia, the HIF-1α subunit becomes stable and regulates the expression of target genes [15].

The rat random-pattern flap model is well-established in investigating the pathophysiology of perfusion and flap survival. The random-pattern flap is harvested without any regard to any known blood supply other than the subdermal plexus [16]. To reduce the risk of distal flap necrosis, a rigid length-to-width ratio of 2:1 or 3:1 should be considered. One of the first experimental random-pattern flap designs was introduced by McFarlane (1965). The cranial-based rectangular flap was designed to become necrotic when harvested in a single-stage procedure. McFarlane compared this control group to the “delay” group, in which only the long axis of the flap was incised, and the flap was returned to the wound bed. After a delay of two weeks, the flap was harvested completely. Flaps of the delay group had significantly reduced necrosis compared to the control group [17,18]. Many modifications have been described, for example, the one of Adamson, who changed the pedicle from cranial to caudal [19,20]. So far, this flap model has been used in studies to compare flap survival after different drug applications [19,21,22]. The main advantages of this flap are that the operation is easy to perform, repeatable, and predictable, and it allows for observation of the flap over a certain period of time [18].

To evaluate the influence of irradiation on flaps, different animal models have been established. Alongside the previously described McFarlane flaps [23], Luginbuhl et al. and Angelos et al. irradiated rats’ belly and harvested axial rotational flaps pedicled on the inferior epigastric vessels [24,25]. In contrast to flaps that are pedicled on specific vessels, the harvest of random-pattern flaps might be easier and faster, which could result in shorter operation times. This is one of the reasons why this model was chosen.

A previous study by our group has shown that preoperative fractional irradiation with a lower individual dose but a higher total dose has a more negative impact on flap perfusion compared to higher single-stage irradiation. Furthermore, postoperative irradiation with 30 Gy was the lethal dose in our setting, with a weight loss of more than 20% over several weeks [22]. In contrast to the damaging side effects of irradiation, low-dose gamma-ray irradiation has been shown to be effective in the acceleration of wound healing [26]. Furthermore, low dose irradiation might have beneficial effects on fracture healing [27]. In a mechanistic study on normal tissue damage in radiotherapy, the mean dose correlated most strongly with the outcome for a parallel organ and the maximum dose for a serial organ [28]. Due to a lack of mechanistic studies on irradiation of skin flaps, the irradiation regimen in this study was orientated on previously described studies [2,22,24,25]. Nevertheless, this study concentrates on the possible negative side effects of irradiation. Therefore, higher irradiation doses were applied.

The hypothesis of this study was that the irradiation of random-pattern flaps with different high doses influences random-pattern flaps negatively, resulting in increasing areas of flap necrosis compared to a non-irradiated control group. We suspect that irradiation with high doses results in the increasing inflammation of the flaps and suppresses angiogenesis compared to non-irradiation. Moreover, we hypothesize that postoperative irradiation influences these parameters more negatively compared to preoperative irradiation. The results of this study should help to increase therapeutic safety in tissue reconstruction, taking into account the possible negative side effects of irradiation.

## 2. Materials and Methods

A total of 25 rats were included in the study and divided in five different treatment groups (n = 5 per group). As previously described, two modified caudally based McFarlane flaps were harvested at the rat’s back with a length of 6 cm and a width of 1 cm [22]. All operations were performed by the same person. Anesthesia was performed using Isofluran. For analgesia, the animals received butorphanol (0.05–0.2 mg per kg) and meloxicam (1 mg per kg). The flaps were located parallel and 1 cm lateral to the spine. For harvest, an incision along their medial, lateral, and cranial side was performed so that their caudal base was 1 cm cranial of the spina iliaca posterior superior. The dissection was deep to the panniculus carnosus and superficial to the deep fascia. After harvest, the flaps were reinserted to their beds and sutured using monofilament sutures. The rats received an antibiotic treatment with enrofloxacin (7.5 mg per kg) for 5 days. Five rats were not irradiated, forming the control group. The right flap of the treatment groups received pre- or postoperative irradiation, while the left flap served as a non-irradiated intraindividual control. Ionizing radiation was generated via an X-ray tube with 150 kV accelerating voltage and a filtering of 2 mm aluminum. The dose rate was 1.56 Gy per minute. In performing the irradiation procedure, the rats received intramuscular anesthesia using ketamine (100 mg per kg) and medetomidine (0.2 mg per kg). The rats were positioned on the belly and transferred to the irradiation unit in a closed isolation cage to protect the rats from pathogens. An area of 7 × 2 cm^2^, including the right flap or the area of the prospective right flap in the case of preoperative irradiation, was irradiated. The fields were limited to the appropriate range with lead collimators. The study was approved by the ethics committee of the government of Middle Franconia (RUF-55.2.2-2532-2-1275-15).

**Figure 1:**
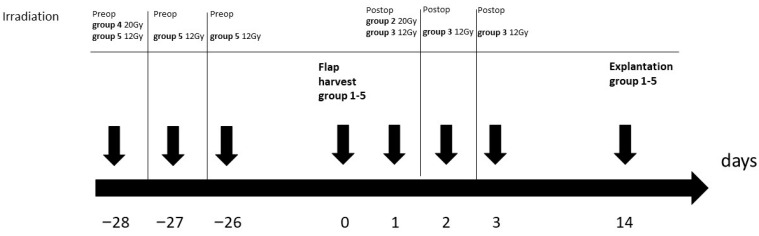
Study design.

Five different treatment groups were examined (Figure 1).

**Figure 2:**
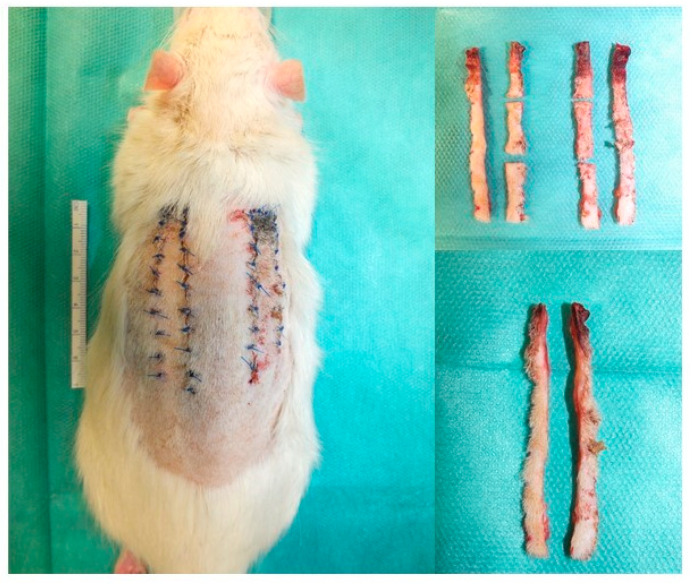
Flap harvest showing the longitudinal division of each flap in halves and each half in thirds, embedding the distal third showing all layers from the dorsal to the basal.

Group 1 served as a non-irradiated control group. Group 2 received postoperative single-stage irradiation with 20 Gy on the first postoperative day, and group 3 received fractional irradiation with 3 × 12 Gy on the first, second, and third day after the surgery. Group 4 received preoperative irradiation with 20 Gy 4 weeks before the surgery, and group 5 received fractional preoperative irradiation with 3 × 12 Gy on the days 28, 27, and 26 before the surgery. Two weeks after the surgery, both left and right flaps were explanted. The quite-high irradiation doses were chosen to examine the damaging effects of irradiation. Each flap was first divided longitudinally and divided into thirds. The cranial lateral third was used for staining, and the cranial medial third for PCR analysis (Figure 2).

For staining, the flap was embedded in such a way that it can be examined in all layers from dorsal to basal.

Hematoxylin and eosin (H&E) staining (Sigma-Aldrich Corporation, St. Louis, MO, USA) and immunohistochemical staining with CD68 (ED1 clone, Bio-Rad, Feldkrichen, Germany) as a marker for macrophages and ERG (clone EP111, Epitomics, Inc., Burlingame, CA, USA) as a vascular marker were performed. PCR analyaes were performed for detection of Interleukin 6, HIF-1α, and VEGF. The NCBI gene database was used for the primer design. GAPDH served as the housekeeping gene. All primers (Table 1) were purchased from the Sigma-Aldrich Corporation. PCR was performed in triplicate of each specimen.

The stainings were analyzed manually and using QuPath 0.2.3 [29]. Histopathological analysis was performed by two people who did not operate on the animals to reduce a possible subjective influence on the results.

The statistical analysis was performed using Microsoft Excel (Microsoft, Redmond, WA, USA) and Prism 9 (GraphPad Software, San Diego, CA, USA). The normal distribution was identified graphically using QQ plots. For the comparison of the necrosis of the CD68+ cells and ΔΔCt in different groups, an ordinary one-way ANOVA following the Tukey test was used. The level for statistical significance was set at *p* < 0.05. Calculating 2-ΔΔCt revealed the relative expression of the control group.

## 3. Results

All animals tolerated the operative procedure without complications. Postoperative irradiation resulted in weight loss of approx. 10% (group 2: weight day at 0 322 ± 20.3 g, weight at day 14 297 ± 17.2 g; group 3: weight at day 0 329 ± 17.8 g, weight at day 14 296 ± 14.2 g). The weight of the rats in groups 1, 4, and 5 remained stable.

**Figure 3:**
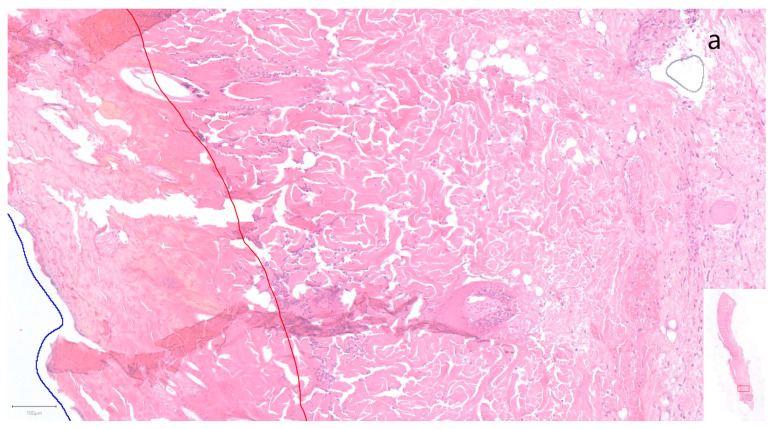

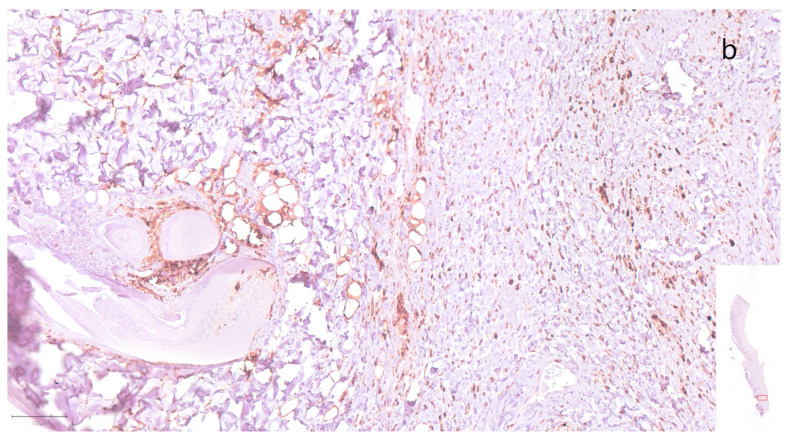

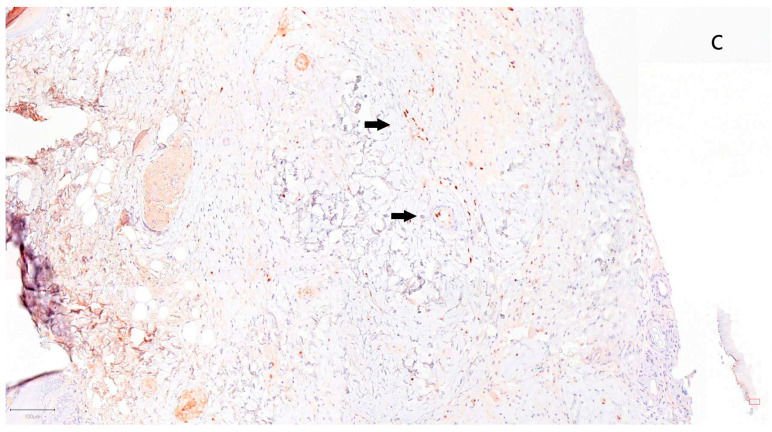
Stainings with H&E ((**a**) necrotic area marked left of the red line, blue line superficial end of the flap), CD68 (**b**), and ERG ((**c**) arrow pointing to the vessels) of a right flap of treatment group 3 (postoperative fractional irradiation with 3 × 12 Gy). Arrows pointing to vessels.

Staining showed all layers of the flap from basal (right part of the image) to dorsal (left parts) (Figure 3, irradiated flap with 3 × 12 Gy postoperative).

**Figure 4:**
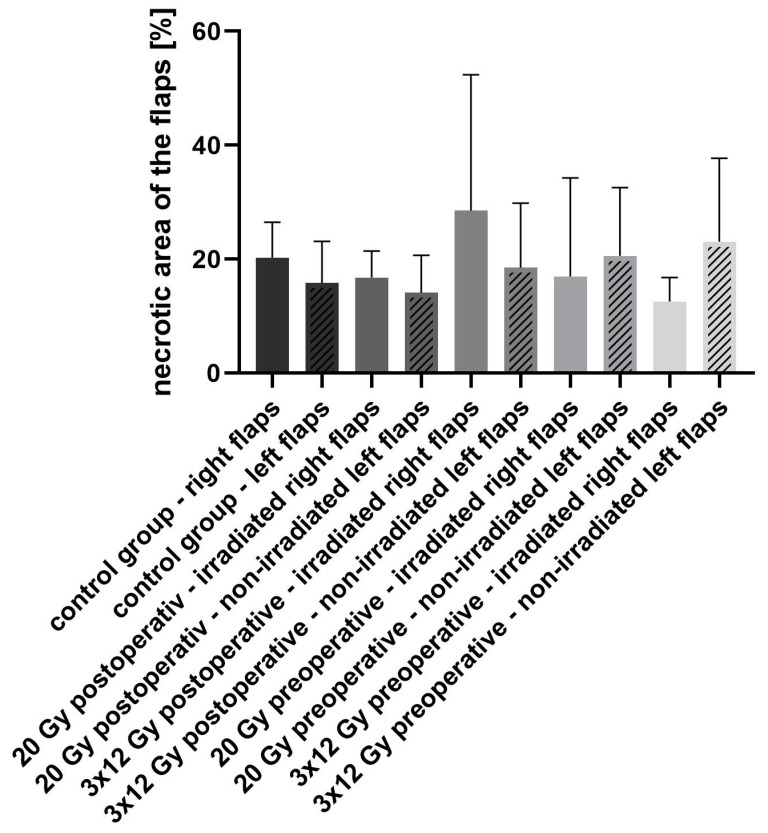
Necrotic area of flaps (%) in all groups without statistically significant differences. However, fractional postoperative irradiation with a total higher dose but lower single doses compared to single-stage irradiation seems to have had a higher influence on the development of necrotic areas.

The necrotic area (Figure 4) of the right flaps—irradiated in groups 2–5—was analyzed via H&E staining.

In the non-irradiated control group (group 1) the mean necrotic area was 20.29% (standard deviation (STD) ± 6.163), 16.80% (STD ± 4.632) in group 2 (postoperative irradiation with 20 Gy), 28.54% (STD ± 23.85) necrotic area in group 3 (fractional postoperative irradiation with 3 × 12 Gy), 16.99% (STD ± 17.31) in group 4 (preoperative irradiation with 20 Gy) and 12.57% (STD ± 4.203) in group 5 (preoperative fractional irradiation with 3 × 12 Gy). There was no statistical difference neither between the different treatment groups nor between the right and left flaps of each group (*p* values of comparison of the rights flap group 1 to group 2: *p* > 0.9999, group 1 to group 3: *p* = 0.9863, group 1 to group 4: *p* > 0.9999, group 1 to group 5: *p* = 0.9914). However, the largest area of necrotic tissue was found in the right flaps after fractional postoperative irradiation with 3 × 12 Gy (group 3). The necrotic areas were only in the superficial parts of the flaps.

**Figure 5:**
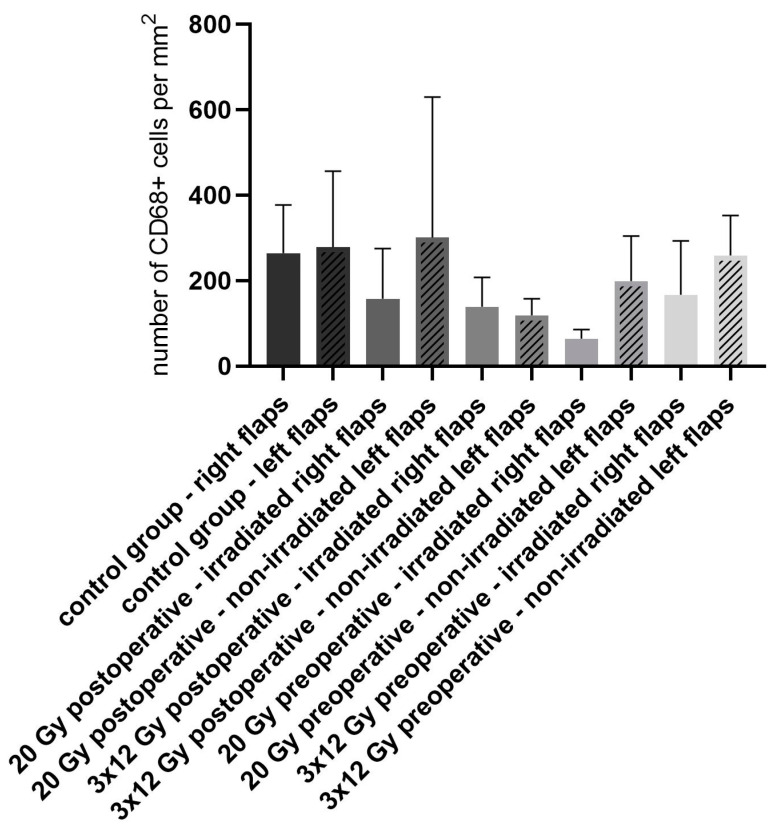
Number of CD68 stained cells per mm^2^ of the flaps in all groups with no statistically significant differences but with lower levels of monocytes and macrophages in the irradiated groups compared to the non-irradiated control group.

The mean number of CD68 stained cells per mm^2^ of the right flaps, that were irradiated in the groups 2–5 (Figure 5), was 264.5 (STD ± 112.7) in group 1 (non-irradiated control group), 157.6 (STD ± 117.5) in the 20 Gy postoperative irradiated flaps (group 2), 139.1 (STD ± 21.22) in the 3 × 12 Gy postoperative irradiated flaps (group 3), 64.12 (STD ± 21.22) in the 20 Gy preoperative irradiated flaps (group 4) and 166.9 (STD ± 126.0) in the 3 × 12 Gy preoperative irradiated flaps (group 5).

There was no statistical difference between the different treatment groups or between the right and left flaps of each group (*p*-values of group 1 compared to group 2: *p* = 0.9728; group 1 to group 3: *p* = 0.9283; group 1 to group 4: *p* = 0.4769; group 1 to group 5: *p* = 0.9850). Compared to the control group, all right flaps in the irradiated groups had lower levels of CD68-stained cells.

**Figure 6:**
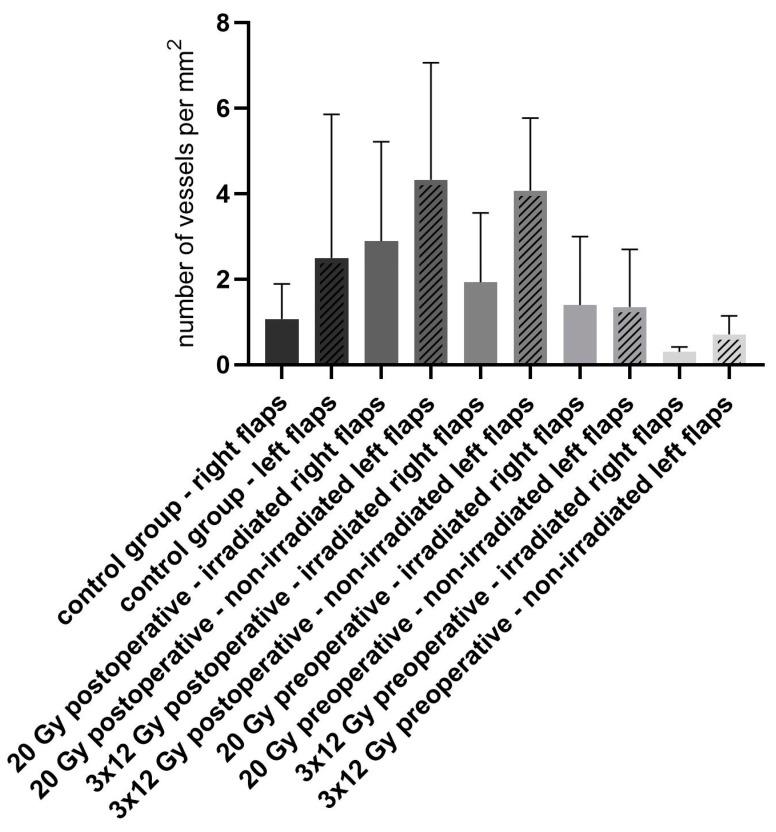
Number of vessels per mm^2^ of the flaps showing no statistically significant differences but a tendency towards increasing the number of vessels in the irradiated flaps, as seen in groups 2, 3, and 4 compared to the control group.

The mean number of vessels per mm^2^ of the right flaps that were irradiated in groups 2–5 (Figure 6), shown via ERG staining, was 1.1 (STD ± 0.817) in group 1 (non-irradiated control group), 2.9 (STD ± 2.327) in group 2 (irradiation with 20 Gy postoperative), 1.9 (STD ± 1.623) in group 3 (irradiation with 3 × 12 Gy postoperative), 1.4 (STD ± 1.6) in group 4 (irradiation with 20 Gy preoperative), and 0.3 (STD ± 0.113) in group 5 (irradiation with 3 ×12 Gy preoperative).

There was no statistical difference between the different treatment groups or between the right and left flaps of each group (*p*-values of comparison between right flaps, group 1 to group 2: *p* = 0.8777; group 1 to group 3: 0.9992; group 1 to group 4: *p* > 0.9999; group 1 to group 5: *p* = 0.9997). The number of vessels in the irradiated right flaps was lower in all groups compared to the non-irradiated left flaps.

**Figure 7:**
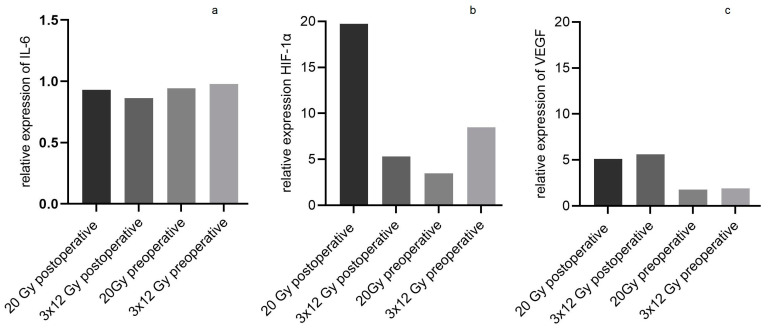
Relative expression of IL-6 (**a**), HIF-1α (**b**), and VEGF (**c**) in the right flaps of all groups showing nearly constant expression of IL-6 in all groups and an increase in VEGF and HIF-1α expression, albeit without statistically significant differences.

The relative expression of IL-6 was 0.93 in group 2 (irradiation with 20 Gy postoperative), 0.86 in group 3 (irradiation with 3 × 12 Gy postoperative), 0.94 in group 4 (irradiation with 20 Gy preoperative), and 0.98 in group 5 (irradiation with 3 × 12 Gy preoperative). The relative expression of HIF-1α was 19.7 in group 2, 5.3 in group 3, 3.5 in group 4, and 8.5 in group 5. The relative expression of VEGF was 5.1 in group 2, 5.6 in group 3, 1.8 in group 4, and 1.9 in group 5 (see all results in Figure 7). There was no statistical difference between the different treatment groups nor between the right and left flaps of each group.

## 4. Discussion

Even though no statistically significant difference was found between the different groups, fractional postoperative irradiation with a higher total dose but lower single doses compared to the single-stage irradiation seems to have a higher influence on the development of necrotic areas in the superficial part of the flaps, as seen via the HE staining. When comparing the necrotic area of the irradiated flaps macroscopically, it has been shown that preoperative fractional irradiation with a lower individual dose but a higher total dose has a more negative impact on flap perfusion compared to higher single-stage irradiation [22].

Comparable to our results, irradiation with a dose of 10 Gy before or after flap harvesting from a rat´s dorsum had no significant effect on flap survival. In that study, flap survival was evaluated clinically and by using a fluorescent dye [30]. Similar results were described by Virolainen et al. They showed that postoperative irradiation with 20 Gy in the rat model did not affect free skin flap survival and showed just minimal histomorphological changes in the irradiation group compared to the non-irradiated control group [31].

As seen in our study, irradiation of rabbit hindlimbs with up to 30 Gy resulted in local hair loss [32]. No skin necrosis was observed. Irradiation caused hypoxia in the subcutaneous tissue relative to the irradiation dose measured on day 1 and 5 weeks after irradiation. Histologic changes such as fibrosis and edema were seen 5 weeks after irradiation. Eleven weeks after irradiation, revascularization of the subcutaneous tissue was observed; nevertheless, fibrosis and vascular changes were seen as signs of chronic irradiation damage [32].

In general, there might be variability in surviving area of the flap, which has been described to be between 22 to 52% [20]. This variability might be explained by a possible nutrient supply available from the original bed to the flap via osmosis, direct vascularization, or neo-vascularization [20]. Moreover, the distal part might survive as a graft when it has direct contact to the underlying wound bed [33]. Separating the random-pattern flap from its wound bed for more than 24 h resulted in significantly more necrosis of the flap compared to the flaps that were separated for 3–12 h from their wound bed [34]. In this study, we did not separate the flap from the wound bed by using plastic sheets as previously described to avoid the development of previously described infections or foreign body reaction [20,33].

CD68 staining for monocytes/macrophages as signs of tissue inflammation did not show differences between the different groups. Interestingly, we measured lower levels of monocytes and macrophages in the irradiated groups compared to the non-irradiated control group, albeit without a statistically significant difference. As previously described, the damaging side effects of irradiation of the skin can be divided into acute and chronic changes [35]. Acute side effects occur immediately or within weeks after irradiation and are reversible when the dose is limited. In contrast, late side effects occur months later [36]. Late side effects are modulated by chronic inflammation, tissue fibrosis, and hypoxia [36,37]. The early phases of fibrogenesis after irradiation result in an upregulation of pro-inflammatory cytokines such as tumor necrosis factor-α (TNFα), interleukins 1 and 6, and many growth factors in the irradiated tissue. Furthermore, chemokines are released and recruit inflammatory cells from the surrounding tissue into the irradiated tissue [37]. An explanation for our findings might be that the specimens were taken 2 or 6 weeks after irradiation, and that this period was too short for the development of the late side effects. Furthermore, the high level of macrophage/monocyte infiltration in the control group might be explained by the reaction of the healthy, non-irradiated tissue to the operation. The operation itself induces an inflammatory response of the tissue which results in the infiltration of inflammatory cells [38]. This physiological response might be higher in healthy tissue compared to damaged irradiated tissue.

In our study design, irradiation has a tendency towards increasing the number of vessels as seen in groups 2, 3, and 4 compared to the control group. Irradiation might have resulted in malperfusion, and this damage, as well as the inducing inflammation, might have induced neo-vascularization as a reaction to these changes. While high-dose irradiation with 30 Gy or 40 Gy seems to have a negative influence on vascularity [2,24,25], the influence of low-dose irradiation has already been described. Whole body single dose irradiation with 1 Gy pre- or postoperatively in rats resulted in a higher number of vessels in Mc Farlane flaps compared to the non-irradiated control group [39]. This might be a result of the positive effect of low-dose whole-body irradiation on the proliferation of normal cells and the stimulation of their defense system [40]. In contrast, intraindividual comparison between the right and left flaps of each treatment group showed a lower vessel number in the irradiated flaps. However, since some irradiation of the left half of the back (for example, via micromovements due to respiratory excursion) cannot be excluded, the intraindividual comparison appears to be less suitable for evaluation. Furthermore, the influence of irradiation on non-irradiated areas as non-targeted bystander or distant effects cannot be excluded [41]. It has been shown that cells that were exposed to ionizing radiation can release signals that induce very similar effects in non-irradiated neighboring cells. These signals are transmitted to the neighboring cells via intercellular gap–junction communication or are released outside the cell or if the cells are cultured into the medium [42]. The bystander effect in this context refers to the detection of responses in unirradiated cells that can reasonably be assumed to have occurred as a result of the exposure of other cells to radiation [43].

Even though the relative expression of VEGF and HIF-1α did not show statistically significant differences between the different treatment groups, irradiation, in general, resulted in an increase in VEGF and HIF-1α expression. This supports the hypothesis that proangiogenic processes are triggered by irradiation to repair the radiation damage in cases when the irradiation damage did not result in cell apoptosis. It is possible that the irradiation doses applied in our study may not have resulted in final cell apoptosis and thus induced neo-vascularization. This effect of irradiation has been described in different oncological studies [44,45]. The fact that the application of VEGF in ischemic wounds in a dermal ulcer rabbit model improved the deficit in wound healing produced by ischemia supports this hypothesis [46]. The expression of IL-6 was nearly constant in all groups, and the irradiation decreased its expression just minimally. One reason might be that IL-6 is synthetized immediately as a reaction to tissue injury. Explantation of the flaps and PCR analysis were performed 2 weeks after the flap harvest. It might be possible that due to the time distance from the injury, parts of the produced IL-6 had already been degraded. According to other studies, irradiation can increase or decrease IL-6 expression; so, in general, it seems that irradiation influences the gene expression of IL-6 just slightly [47,48].

One limitation of this study is the small number of animals per group (n = 5), which might be a reason for the non-statistically significant differences that we have found. High differences of the values have a higher influence within a small group size. In contrast to perforator flaps, these flaps are randomly perfused, so there might be variation rates among individuals [49]. Nevertheless, the results showed some tendencies that might indicate the negative influence of irradiation in a random-pattern flap model. To determine some possibly positive side effects, further experiments involving irradiation with lower doses might be useful. In addition, the short intervals between irradiation and surgery or vice versa are transferable to actual treatment in human patients only to some extent. In order to enable even better transferability into clinical application, longer intervals between surgery and radiation should be considered. Months or years between radiation and surgery, or vice versa, do not appear to be adequate in the animal model, as some trends have demonstrated in this study design.

Knowledge about the influence of different irradiation doses and regimens on flap survival are not only useful in defect reconstruction in plastic surgery; they might have an influence on future possibilities in tissue engineering; for example, Ɣ-radiation sterilization improved mechanical and thermal properties of polymer matrix [50]. Nevertheless, the application of tissue-engineered constructs in reconstructive surgery has not been standardized yet.

## 5. Conclusions

Postoperative cumulative higher doses of irradiation appeared to result in an increase in necrosis, especially in the superficial layers of the flap, compared to preoperative or single-stage irradiation. Moreover, the irradiated flaps showed an increased expression of VEGF and HIF-1α, possibly as reaction to hypoxia. Furthermore, the irradiated flaps showed lower numbers of vessels in the intraindividual comparison, which might indicate a depression of neo-vascularization. Further experiments are necessary in order to receive statistically significant differences.

## Figures and Tables

**Table 1:** qPCR primer sequence.

Gene	5′-3′ Primer Sequence
GAPDH	For: GAAGGTCGGTGTGAACGGAT Rev: TGAACTTGCCGTGGGTAGAG
Interleukin 6	For: GACTTCCAGCCAGTTGCCTT Rev: GCAGTGGCTGTCAACAACAT
HIF-1α	For: GCAACTGCCACCACTGATGA Rev: GCTGTCCGACTGTGAGTACC
VEGF	For: AATGATGAAGCCCTGGAGTG Rev: ATGCTGCAGGAAGCTCATCT

## Data Availability

The data presented in this study are available on request from the corresponding author.
